# A New Family of Cumulative Indexes for Measuring Scientific Performance

**DOI:** 10.1371/journal.pone.0047679

**Published:** 2012-10-31

**Authors:** Marcin Kozak, Lutz Bornmann

**Affiliations:** 1 Department of Quantitative Methods in Economics, Faculty of Economics, University of Information Technology and Management in Rzeszow, Rzeszów, Poland; 2 Division for Science and Innovation Studies, Administrative Headquarters of the Max Planck Society, Munich, Germany; University of Illinois-Chicago, United States of America

## Abstract

In this paper we propose a new family of cumulative indexes for measuring scientific performance which can be applied to many metrics, including h index and its variants (here we apply it to the h index, h(2) index and Google Scholar's i10 index). These indexes follow the general principle of repeating the index calculation for the same publication set. Using bibliometric data and reviewer scores for accepted and rejected fellowship applicants we examine how valid the cumulative variant is compared to the original variant. These analyses showed that the cumulative indexes result in higher correlations with the reviewer scores than their original variants. Thus, the cumulative indexes better reflect the assessments by peers than the original variants and are useful extensions of the original indexes. In contrast to many other measures of scientific performance proposed up to now, the cumulative indexes seem not only to be effective, but they are also easy to understand and calculate.

## Introduction

In a scientific world of limited financial resources, easy-to-handle bibliometric metrics are highly desirable for evaluation and comparison purposes. Thus, it is not surprising that the h index has immediately attracted great interest amongst the public [1]. Hirsch [2] proposed the h index as a criterion to quantify the scientific output of a single scientist. Hirsch's [2] index depends on both the number of a scientist's publications and their impact on his or her peers: “A scientist has index *h* if *h* of his or her *N_p_* papers have at least *h* citations each and the other (*N_p_*−*h*) papers have fewer than ≤*h* citations each” (p. 16569). The h index is intended as an alternative to other citation-based indexes that could be used to measure research performance (e.g. the number of highly cited papers published, the mean number of citations per paper and the total number of citations). The main advantage of the h index is that it “represents an attempt to strike a balance between productivity and quality and to escape the tyranny of power law distributions which place strong weight on a relatively small number of highly cited papers” (See [3], p. 377).

The metric proposed by Hirsch [2] has received a lot of attention not only from the public but also from bibliometric research. According to Zhang et al. [1], Hirsch [2] “had launched a new research direction in informetrics and scientometrics” (p. 583). In recent years some literature surveys were published which summarize the extensive research on the h index [1], [4], [5]. The h index research frequently deals with its advantages and disadvantages, its properties and its field of application. Although this research is still an on-going hot topic in bibliometrics [6], the h index is already a widely accepted metric and was included as an indicator in Web of Science (Thomson Reuters) and Scopus (Elsevier) within two years of its publication [7].

Based on the identified disadvantages of the h index (for example, a scientist's h index depends on the person's scientific age and a higher h index can be expected in some areas of research than in others), a number of different h index variants have been proposed. For a meta-analysis of studies reporting correlations between the h index and different h index variants, Bornmann et al. [8] were able to research 37 variants. For example, the g index proposed by Egghe [9] places more weight on the citation performance of a set of papers (by focussing on the highly-cited papers) than the h index does. Although several h index variants have been suggested, mathematical improvement alone cannot increase the validity of these variants against the original index. One must be able to ask how valid the variant is (compared to the h index). One common approach is to compare a metric against independent performance evaluations (expert evaluations), at best for a broad range of scientists with different degrees of productivity [10], [11], [12].

The aim of this paper is to propose a new family of cumulative indexes for measuring scientific performance. These indexes follow the general principle of repeating the index calculation for the same publication set. This repetition can be carried out with different data sets (scientists, journals etc.) and with various indexes (here: h index, h(2) index and Google Scholar *i*10 index). We present a general algorithm for the new family of indexes, and validate the cumulative index variant against its original (h index, h(2) index and Google Scholar *i*10 index) by using reviewer scores for fellowship applicants.

## Materials and Methods

### Data set

To validate the indexes against assessments by peers, a data set of applicants to the Young Investigator Programme (YIP) of the European Molecular Biology Organization (EMBO, Heidelberg, Germany) was used [13], [14], [15], [16]. The YIP has been supporting outstanding young group leaders in the life sciences in Europe since 2000. The selection committee of the YIP consisted of ten EMBO members. The evaluation procedure for applicants comprises of an interview with an EMBO member expert in the area of the applicant's research and an evaluation by all members of the programme's selection committee. Each committee member individually evaluates the applicant and their research, taking into account the interviewer's report, and assigns a score between 1–10, with 10 being the best score. All applications are ranked according to their average score and decisions about approval or rejection are made after debate at a committee meeting.

The study involves 288 applicants to the YIP, of which 39 were approved and 249 rejected, from 2001 and 2002. The 288 YIP applicants included in this study published a total of 5,891 papers (articles, letters, notes, and reviews) prior to application (publication window: 1984 to the application year, 2001 or 2002). These papers received an average of 46.48 citations (citation window: from publication year until 2007). The bibliographic data of the applicants' papers were taken from the Web of Science database and were double-checked in the Medline database (provided by the National Library of Medicine, Bethesda, MD, USA) and with the applicants' lists of publications [13].

### Indexes compared

The following original indexes are compared in this paper with their cumulative variants: (1) the h index as defined above; (2) Google Scholar's *i*10 (see http://scholar.google.com/citations) indicates the number of papers which have at least ten citations (the index is a simple example of scoring rules [17]); (3) the h(2) index “is defined as the highest natural number such that his h(2) most-cited papers each received at least [h(2)]^2^ citations” [18]. The three corresponding cumulative indexes are named as follows: cum h, cum h(2), and cum *i*10. All index variants are compared to the reviewer scores.

### A general algorithm for a family of cumulative indexes

Consider an index *T*; to construct a cumulative index *T* of order *k*, that is, cum *T_k_*, use the following algorithm:

1. Sort papers by number of citations (or prepare the data in a way index *T* requires)
2. Determine index *T*.
3. From the list of papers and their citations remove those citations that have been used to determine index *T* in this iteration.
4. Repeat steps 1–3 (*k*−1) times.
5. Determine the cumulative index as 
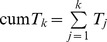
.

Below are two examples of the above general algorithm, adjusted to the h index and Google Scholar's *i*10 index.

### Algorithm for computing the cum h index of order k (cum h_k_) is as follows

1. Sort papers by number of citations.
2. Determine *h*.
3. From the top *h* papers remove *h* from their citations.
4. Repeat steps 1–3 (*k*−1) times.
5. The cumulative index of order *k*, that is cum *h_i_*, is 
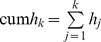

The algorithm for computing the cum h(2) index is very similar, and differs only in that in step (1) the number of citations is squared; the rest remains unchanged.

### Algorithm for computing the cum i-j (cum i10 in particular is obtained for j = 10) index of order k (cum ij_k_) is as follows

1. Sort papers by number of citations.
2. Determine *j* index (a number of papers that have at least *j* citations).
3. From the top *w* papers remove *w* from their citations.
4. Repeat steps 1–3 (*k*−1) times.
5. The cumulative index of order *i*, that is cum *i-j_k_*, is 
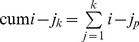

In this paper, we calculate the cumulative version of order 10 for each index.

Besides the different indexes and cumulative variants, we include for each applicant the number of papers and the total citation counts in the analysis.

### Data analysis

Associations among the indexes and the reviewer scores are determined by Spearman's rank-order correlation [19].The data are analyzed with R [20]. In contrast to many other measures of scientific performance proposed up to now, the cumulative indexes are easy to calculate. The R codes for the indices used in this paper are given in the Appendix S1 in a supplementary file.

## Results

### Spearman's rank-order correlations

Spearman's rank-order correlations among the indexes (h, cum h_10_, h(2), cum h(2)_10_, *i*10, and cum *i*10_10_), the number of papers, total citation counts, as well as the reviewer scores are presented in Table 1. The coefficients for the correlations between the different metrics and the reviewer scores are in a range of r = .15 (number of papers) and r = .47 (cum *i*10_10_). If we compare these coefficients with rank-ordered correlations between the average number of citations and Research Assessment Excellence scores in the year 2001 [21], the coefficients in Table 1 are lower than most of the coefficients published by Mahdi et al. [21] for biomedical sciences. However, there seems to be a difference between output and impact oriented metrics: Whereas in Table 1 the number of papers correlates weakly with the reviewer scores (r = .15), the correlation between total citation counts and reviewer scores is comparably strong (r = .41). The comparably high quality of total citation counts for measuring scientific performance could also be shown in other studies. For example, Bensman and Wilder [22] concluded on the basis of validation studies that the prestige of journals in chemistry is correlated with the total number of citations stronger than with the Journal Impact Factors [23] of the journals.

**Table 1:** Spearman's rank-order correlations between the different indexes and the reviewer scores (*n* = 288).

	no. of papers	no. of cites	*h*	cum *h*_10_	*h*(2)	cum *h*(2)_10_	*i*10	cum *i*10_10_
no. of cites	0.57	1.00						
*h*	0.75	0.86	1.00					
cum h_10_	0.63	0.95	0.91	1.00				
h(2)	0.94	0.72	0.89	0.78	1.00			
cum h(2)_10_	0.86	0.81	0.97	0.87	0.96	1.00		
*i*10	0.84	0.79	0.96	0.86	0.95	0.99	1.00	
cum *i*10_10_	0.46	0.94	0.82	0.97	0.63	0.74	0.73	1.00
**Reviewer scores**	**0.15**	**0.41**	**0.39**	**0.43**	**0.25**	**0.33**	**0.33**	**0.47**

Most of the indexes in Table 1 are very strongly correlated (within the range of r = .8 and r = .99). Although the proposed indexes may be conceptualized differently, they could be called redundant in empirical application. However, the index Google Scholar's cum *i*10_10_ seems to be an exception: it has the weakest correlation with most of the other indexes. Since this index is correlated strongest with the reviewer scores (r = .47), this index reflects at best – compared to the other indexes – the expert evaluations by the reviewers. Furthermore, it is interesting to see that the cumulative version of each of the compared indexes is clearly stronger correlated with the reviewer scores than their non-cumulative counterparts. The greatest increase is observed for Google Scholar's *i*10 index (from r = 0.33 to r = 0.47).

### Advantages and disadvantages of h index variants and other metrics

Although there seems to be a general advantage of the cumulative indexes over the original, the advantage of a certain cumulative variant also depends upon the index selected as the base one. In general, depending on this choice, an index can be created so that papers with fewer citations (which are frequently ignored by metrics) are also taken into account (which can be very important for junior scientists) or that high-impact papers have a greater weight than those with fewer citations (which is important especially for senior scientists). These two features are examples of disadvantages of the h index, which hardly distinguishes between young scientists who have published, say, 4–5 papers, and might not differentiate between two senior scientists, with say 40–50 papers, one of whom has several high-impact papers while the other has not: both can have the same h index. In general, the h index ignores papers with smaller citations than h and all citations over h. The cum h_i_ index, for example, between two scientists with the same number of papers and the same h index will favor the one who has more high-impact papers.

Google Scholar's *i*10 index (or, in general, an *i*-*j* index, which counts the number of articles with at least *j* citations) is extremely easy to understand, apply and interpret. As we show in this study, the original, but especially its cumulative version, seems to be efficient at recording scientific performance. However, for a concrete evaluation study, the index citation thresholds should be adjusted properly and only scientists of almost the same age and from the same field should be compared. If we take a smaller *j*, say *i*5 or even *i*3, we can apply it for junior scientists at early stages of their careers; employing the cumulative index, say cum *i*3_5_ or cum *i*5_5_, we would be able to come up with higher discrimination of the scientists. For example, for three scientists with 4 published papers each and with citations A = (3, 3, 2, 1), B = (7, 5, 3, 1) and C = (12, 7, 3, 0), we will have *i*3, cum *i*3_5_ and cum *i*5_5_ indexes as follows:
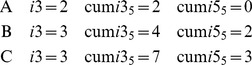
A similar index can be used to assess the performance of top-tier scientists, for example by constructing a cum *i*50_10_ index. A paper having 500 citations or more will add 10 to the cum *i*50_10_ index, so will have 10 times more weight than a paper with citations from 50 to 99. The efficiency of cum *i-j_k_* index is also proved in this paper by the relatively high correlation of cum *i*10_10_ with the reviewer score.

Waltman and van Eck [24] reported inconsistencies with the h index, which in their opinion are sufficiently significant to claim that the h index should not be used to assess individual scientists. Here we report a problem with the cum h_i_ index that may be due to similar inconsistencies to those reported by Waltman and van Eck [24]. If we consider two scientists both of whom have published three papers, but which gained a different number of citations of (3, 3, 3) for first researcher and (3, 3, 2) for second researcher, than the former researcher will have h = 3 and cum h_2_ = 3 and the latter researcher h = 2 and cum h_2_ = 4, an illogical result. This topic requires additional studies. To some extent it might be attributed to the rule in example 3 by Waltman and van Eck [24] (“If scientist X_1_ is ranked higher than scientist Y_1_ and scientist X_2_ is ranked higher than scientist Y_2_, then a research group consisting of scientists X_1_ and X_2_ should be ranked higher than a research group consisting of scientists Y_1_ and Y_2_”) because applying any cumulative index is somewhat similar to applying it for a research group and summing up the group members. Hence a rule follows that a cumulative index may bear problems of its original index (although does not have to), and this should always be checked when constructing any cumulative index.

## Discussion

In this paper we propose a new family of cumulative indexes for measuring scientific performance which can be applied to many h index variants and other research performance metrics (here: to the h index, h(2) index and Google Scholar's i10 index). Not all indexes have their natural representatives in the cumulative family. An example is the g index [9], for which it is not easy to decide which citations should be removed from the papers used to determine g in the subsequent iterations of the algorithm. One idea is to remove 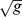 citations from g papers, but this requires further studies.

Using a dataset with bibliometric data of accepted and rejected fellowship applicants we examine in this paper how valid the cumulative variant compared to the original variant is. This examination shows that the cumulative variants lead to higher correlations with the reviewer scores. Thus, the cumulative variants seem to better reflect the assessments by peers than the original variants and thus can be seen as meaningful extensions of the original indexes. Most h index variants and extensions [8] proposed up to now are difficult to calculate and understand, which is not the case with the cumulative indexes – it seems not only to be effective, but they are also relatively easy to understand and calculate for those who understand the corresponding non-cumulative index. The latter features of the cumulative indexes are important, since according to Franceschini and Maisano [25] “indicators that are difficult to understand and interpret, because reference to real data has been ‘lost’, are often rejected by potential users” (p. 495).

## Conclusions

What is important, and what follows from this study, is that the cumulative family of indexes can be a source of well-grounded and commonly used indexes to assess scientist performance. However, owing to its flexibility it can be used to construct indexes for a particular evaluation task, just as the above example of assessing performance of fellowship applicants shows. Different parameters for a cum *i*-*j_k_* index might be used for assessing scientists with different experience or scientific areas, or for different tasks etc. Although an index from the cumulative family seems to be a good selection for an evaluative bibliometric study, we would like to stress that it is not sufficient to use only one index. Scientific work is a complex and multidimensional activity [26], which should be assessed by more than one performance metric and by indicators beyond pure output and impact.

## Supporting Information

Appendix S1 **R code for the indices used in the paper.**
(DOCX)
