# Supplementary material for: A New Family of Cumulative Indexes for Measuring Scientific Performance
Source: PLoS One. 2012 Oct 31;7(10):e47679. doi: 10.1371/journal.pone.0047679 (PMC3485265; doi:10.1371/journal.pone.0047679)
Supplement: Appendix S1 — R code for the indices used in the paper. (DOCX) [file pone.0047679.s001.docx]

Appendix: R code for the indices used in the paper.

Below, x is a vector of citations for a researcher (it does not have to be ordered by size), such as x <- c(0, 34, 12, 13, 14, 0). The functions use the CITAN package and partly bases upon its code.

index.h2 <- function(x) {

if (length(x) == 0) return(0)

if (mode(x) != "numeric") return(NA)

if (any(x < 0)) return(NA)

x <- x[!is.na(x)]

x <- sort(x, decreasing = TRUE)

x <- x^2

index.h(x)

}

index.h2.cum <- function(x, i = 10) {

if (length(x) == 0) return(0)

if (mode(x) != "numeric") return(NA)

if (any(x < 0)) return(NA)

x <- x[!is.na(x)]

x <- sort(x, decreasing = TRUE)

x <- x^2

index.Hi(x, i = i)

}

index.Hi <- function(x, i = 3) {

if (length(x) == 0) return(0)

if (mode(x) != "numeric") return(NA)

if (any(x < 0)) return(NA)

x <- x[!is.na(x)]

x <- sort(x, decreasing = TRUE)

Hi <- vector()

for (j in 1:i) {

Hi[j] <- index.h(x)

x[1:(Hi[j])] <- x[1:(Hi[j])] - Hi[j]

x <- sort(x, decreasing = TRUE)

}

return(sum(Hi))

}

cum.ij <- function(x, i = 10, j = 10) {

if (length(x) == 0) return(0)

if (mode(x) != "numeric") return(NA)

if (any(x < 0)) return(NA)

x <- x[!is.na(x)]

x <- sort(x, decreasing = TRUE)

ij <- vector()

for (k in 1:i) {

ij[k] <- length(which(x >= j))

x[1:ij[k]] <- x[1:ij[k]] - ij[k]

x <- sort(x, decreasing = TRUE)

}

return(sum(ij))

}

library(CITAN)

x <- c(0, 34, 12, 13, 14, 0)

index.h2(x)

index.h2.cum(x, i = 10)

index.h(x)

index.Hi(x, i = 10)

cum.ij(x, i = 10, j = 1)

cum.ij(x, i = 10, j = 10)
